# Efficient Metabolic Fingerprinting of Follicular Fluid Encodes Ovarian Reserve and Fertility

**DOI:** 10.1002/advs.202302023

**Published:** 2023-06-13

**Authors:** Jiao Wu, Chunmei Liang, Xin Wang, Yida Huang, Wanshan Liu, Ruimin Wang, Jing Cao, Xun Su, Tao Yin, Xiaolei Wang, Zhikang Zhang, Lingchao Shen, Danyang Li, Weiwei Zou, Ji Wu, Lihua Qiu, Wen Di, Yunxia Cao, Dongmei Ji, Kun Qian

**Affiliations:** ^1^ Shanghai Key Laboratory of Gynecologic Oncology Department of Obstetrics and Gynecology Renji Hospital School of Medicine Shanghai Jiao Tong University Shanghai 200127 P. R. China; ^2^ State Key Laboratory of Systems Medicine for Cancer School of Biomedical Engineering and Institute of Medical Robotics Shanghai Jiao Tong University Shanghai 200030 P. R. China; ^3^ Division of Cardiology Renji Hospital School of Medicine Shanghai Jiao Tong University Shanghai 200127 P. R. China; ^4^ Department of Obstetrics and Gynecology the First Affiliated Hospital of Anhui Medical University Hefei 230022 P. R. China; ^5^ NHC Key Laboratory of Study on Abnormal Gametes and Reproductive Tract Anhui Medical University Hefei 230032 P. R. China; ^6^ Key Laboratory of Population Health Across Life Cycle Anhui Medical University Hefei 230032 P. R. China; ^7^ Key Laboratory for the Genetics of Developmental & Neuropsychiatric Disorders (Ministry of Education) Renji Hospital Bio‐X Institutes School of Medicine Shanghai Jiao Tong University Shanghai 200240 P. R. China; ^8^ Key Laboratory of Fertility Preservation and Maintenance of Ministry of Education Ningxia Medical University Yinchuan 750003 P. R. China; ^9^ Department of Obstetrics and Gynecology Ren Ji Hospital School of Medicine Shanghai Jiao Tong University Shanghai 200127 P. R. China

**Keywords:** biofluids, biomarkers, diagnostics, mass spectrometry, metabolism

## Abstract

Ovarian reserve (OR) and fertility are critical in women's healthcare. Clinical methods for encoding OR and fertility rely on the combination of tests, which cannot serve as a multi‐functional platform with limited information from specific biofluids. Herein, metabolic fingerprinting of follicular fluid (MFFF) from follicles is performed, using particle‐assisted laser desorption/ionization mass spectrometry (PALDI‐MS) to encode OR and fertility. PALDI‐MS allows efficient MFFF, showing fast speed (≈30 s), high sensitivity (≈60 fmol), and desirable reproducibility (coefficients of variation <15%). Further, machine learning of MFFF is applied to diagnose diminished OR (area under the curve of 0.929) and identify high‐quality oocytes/embryos (*p* < 0.05) by a single PALDI‐MS test. Meanwhile, metabolic biomarkers from MFFF are identified, which also determine oocyte/embryo quality (*p* < 0.05) from the sampling follicles toward fertility prediction in clinics. This approach offers a powerful platform in women's healthcare, not limited to OR and fertility.

## Graphical abstract

This work develops a platform to encode ovarian reserve (OR) and fertility through machine learning of metabolic fingerprinting of follicular fluid (MFFF), using particle‐assisted laser desorption/ionization mass spectrometry (PALDI‐MS). Such MFFF may help to diagnose diminished OR and identify high‐quality oocytes/embryos (*p* < 0.05) by a single PALDI‐MS test. This work would advance the metabolic analysis of gynecological diseases.

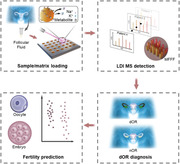

## 1 Introduction

Ovarian reserve (OR) and fertility reflect reproductive potential, which is critical in women's healthcare.^[^ ^1^ ^]^ Precise encoding of OR and fertility is vital in determining the appropriate treatments, affecting 10–32% of women at reproductive age, based on the estimates from the National Society for Assisted Reproductive Technology (SART) system in the United States.^[^ ^2^ ^]^ Current analytical methods in clinics rely on a combination of tests including biochemical analysis and ultrasound imaging.^[^ ^3^ ^]^ These methods are based on the selected protein biomarkers (such as follicle‐stimulating hormone [FSH] and antimüllerian hormone [AMH]) or physical measurements (antral follicular count [AFC]), which cannot serve as a multi‐functional platform with limited information from specific biofluids like follicular fluid (FF).^[^ ^4^ ^]^ Accordingly, a single test that offers comprehensive metabolic information would be desirable in encoding OR and fertility, to engage various clinical applications toward women's healthcare.

The selection of biomarkers from biofluids marks a turning point in improving diagnostics, particularly for OR and fertility.^[^ ^5^ ^]^ In selecting biomarkers, differing from nucleic acids and proteins,^[^ ^6^ ^]^ the metabolites serve as end‐products of pathways, allowing the characterization of biological and pathological processes in real time.^[^ ^7^ ^]^ Notably, most biomarkers of the OR and fertility are hormones (e.g., FSH and AMH) in the blood circulating in the whole body, which affords indirect evaluation by relying on feedback from the pituitary or follicles in the ovary.^[^ ^8^ ^]^ For comparison, FF secreted by follicles in ovarian is directly related to the variation of OR, oocyte developmental competence, and embryo viability.^[^ ^9^ ^]^ Thus, new panels of metabolic biomarkers in FF hold promise for encoding OR and fertility, considering the function of metabolites at the end of pathways. To date, the available analyses using metabolic biomarkers in FF are still preliminary for encoding OR and fertility, only dealing with small cohorts (≈40–150) and single functions like the diagnosis of diminished OR (dOR).^[^ ^9^, ^10^ ^]^ Therefore, new panels of metabolic biomarkers with multi‐functions are needed to be constructed in a well‐defined cohort to improve the diagnostic accuracy and predictive performance, as the next‐generation tool.

Mass spectrometry (MS) is a fundamental technique for metabolic biomarker detection that allows for the measurement of compounds in a label‐free manner.^[^ ^11^ ^]^ Conventional MS techniques using chromatography (e.g., liquid chromatography, LC or gas chromatography, GC) require pretreatment and enrichment of samples (30–60 min/sample) to overcome the high complexity of biofluids and low abundance of metabolites (down to approximately pmol), hindering its widespread applications.^[^ ^12^ ^]^ Importantly, laser desorption/ionization (LDI) MS enables efficient analysis of biofluids with minimal sample pretreatment (approximately min/sample) and high sensitivity (approximately pmol), by using defined matrix materials on the microarray chip to selectively trap metabolites.^[^ ^13^ ^]^ To date, protocols based on LDI MS for metabolic fingerprinting have been utilized to detect a wide range of biofluids (e.g., serum, urine, tear, cerebrospinal fluid, and aqueous humor).^[^ ^5^, ^6^, ^14^ ^]^ Despite this, the protocol using LDI MS for metabolic fingerprinting of FF (MFFF) has not been developed. Moreover, LDI MS‐based metabolic fingerprinting has been applied to investigate a range of diseases such as cancers, infectious diseases, and cardiovascular/cerebrovascular diseases.^[^ ^15^ ^]^ However, the specific application of LDI MS‐based metabolic fingerprinting for encoding OR and fertility using MFFF remains a significant challenge. Therefore, MFFF using LDI MS would offer an efficient platform to characterize OR and fertility, underpinning women's healthcare.

**Scheme 1:**
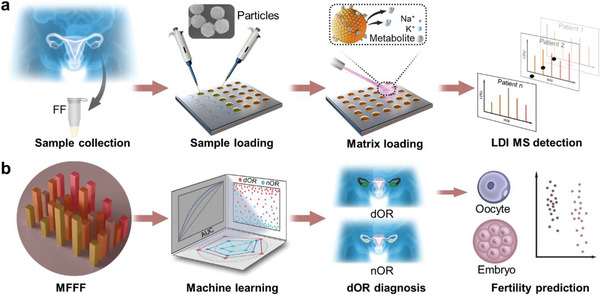
Schematic of MFFF protocol for encoding OR and fertility. The protocol comprised two parts: the acquisition of MFFF based on PALDI‐MS and subsequent analysis of MFFF for encoding OR and fertility. a) For the acquisition of MFFF based on PALDI‐MS, FF samples were first collected from enrolled subjects. Subsequently, ≈100 nL of raw FF samples and 1 µL of particle suspension were loaded onto the microarray chip, followed by the LDI MS detection. MFFF was obtained from the native MS spectra of each subject. b) For the analysis of MFFF, machine learning algorithms were used to achieve a precise diagnosis of dOR. Further, we determined the quality of oocytes/embryos for dOR subjects.

Herein, we performed MFFF from follicles using particle‐assisted laser desorption/ionization mass spectrometry (PALDI‐MS) to encode OR and fertility (**Scheme** **1**). PALDI‐MS allowed efficient MFFF, showing fast speed (≈30 s), high sensitivity (≈60 fmol), and desirable reproducibility (coefficients of variation [CVs] < 15%). Further, we achieved a precise diagnosis of dOR (area under the curve [AUC] of 0.929) and identification of high‐quality oocytes/embryos (*p* < 0.05) using machine learning of MFFF detected by a single PALDI‐MS test. Meanwhile, we identified metabolic biomarkers from MFFF, which also determined oocyte/embryo quality (*p* < 0.05) from the sampling follicles toward fertility prediction in clinics. Therefore, our work would advance the encoding of OR and fertility for women's healthcare.

## 2 Results and Discussion

### 2.1 Metabolite Detection through PALDI‐MS

**Figure 1:**
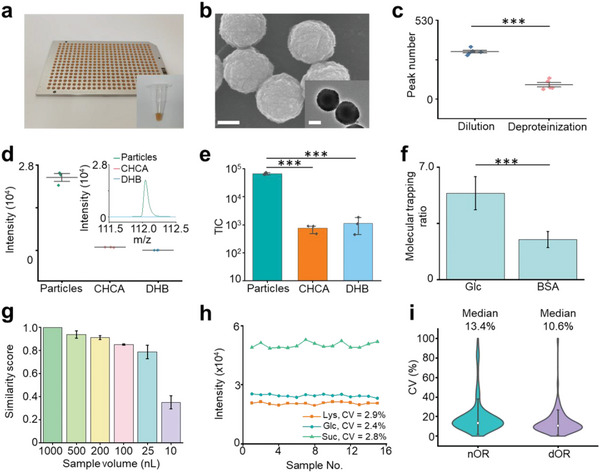
Characterization of the PALDI‐MS. a) Digital images of the microarray chip with 384 sample spots and particle solution dispersed by water in a 600 µL tube for the insert image. The distance between the centers of two spots on the chip was 4.5 mm. b) Scanning electron microscopy (SEM) image and transmission electron microscopy (TEM) image of particles (inset) showing the surface roughness of the particles. The scale bars for SEM and TEM images were 100 nm. c) The comparison for sample pretreatment of dilution and deproteinization, displaying more metabolites detected by dilution treatment. *** represented *p* < 0.001. d) The intensities of metabolites detected using particles, CHCA and DHB as the matrix, and typical mass spectra (insert) of 1.12 nmol Ala. The standard deviation (s.d.) of three tests was obtained as an error bar. e) The TIC of mass spectra for detecting standard mixture (including 10 ng mL^−1^ Glc, Suc, Ala, and Arg) using particles, CHCA, and DHB as the matrix. The error bars represented the s.d. of three replicates. *** represented *p* < 0.001. f) Molecular trapping ratios for Glc and BSA using particles as the matrix through elemental mapping results showing the selective trapping of particles for metabolites. The error bars represented ± s.d. *** represented *p* < 0.001. g) Cosine similarity scores of mass spectra from raw FF and its dilutions with dilution folds of 2–100 using 10–500 nL of raw FF samples. The error bars showed the s.d. of three samples. h) CVs of intensities for a standard mixture containing Lys, Glc, and Suc in 15 tests, showing the high reproducibility of PALDI‐MS using particle as the matrix. i) The CVs of intensities for FF samples from nOR and dOR subjects with five independent tests. The error bars represented ± s.d.

We developed the PALDI‐MS for fast, sensitive, reproducible records of MFFF by utilizing specific particles. The particles were prepared from a solve‐thermal method,^[^ ^12^ ^]^ which obtained about 2 g of products per batch for large‐scale use (capable of 2 × 10^6^ PALDI‐MS tests, inset of **Figure** **1**a). The synthesized particles possessed a polycrystalline structure corresponding to the Fe_3_O_4_ crystal structure (Joint Committee on Powder Diffraction Standards [JCPDS]:99‐0073), as confirmed by X‐ray diffraction (XRD) analysis (Figure S1a, Supporting Information).

For fast speed, we achieved metabolite detection through a microarray chip with automatic scanning mode and direct analysis of samples with minimum pretreatment. We detected the metabolites at a speed of ≈3 s per sample (2 s detection by 2000 shots with the laser frequency of 1000 Hz and 1 s interval among samples), using a 384‐microarray chip through the automatic scanning mode (Figure 1a). Further, we were able to directly conduct PALDI‐MS analysis of biofluid samples due to the use of particles. The particles with a uniform size (polydispersity index: 0.01) of ≈200 nm consisted of nanocrystals (≈4.5 nm), forming rough cavities on the surface to trap small metabolites (Figure 1b and Figure S1b–e, Supporting Information). Moreover, for sample pretreatment, we detected more metabolites in FF through the dilution process (≈10 s) with a peak number of 445, compared to deproteinization treatment (≈15 min) with a peak number of 293 (*p* < 0.05, Figure 1c), due to the loss of metabolites during the deproteinization treatment (Figure S1f, Supporting Information). Importantly, we achieved the minimum sample pretreatment for FF detection with a facile dilution process, owing to the high salt (0.5 m NaCl) and protein (bovine serum albumin, BSA, 5 mg mL^−1^) tolerance of PALDI‐MS (Figure S1g,h, Supporting Information). Accordingly, we demonstrated the fast speed of PALDI‐MS for small metabolite detection within ≈30 s per sample for the whole experiment, through a microarray chip and direct analysis of samples with minimum pretreatment.

For sensitive detection, we assessed the detection performance of PALDI‐MS for small metabolites in standard solution and biofluids. Due to the preferential trapping of small metabolites through particles, the limit of detection (LOD) using particles has remarkably improved by four orders of magnitude (down to ≈60 fmol, Figure 1d and Figure S2 and Tables S1 and S2, Supporting Information) for detecting alanine (Ala), arginine (Arg), glucose (Glc), and sucrose (Suc), compared to the traditional organic matrices such as *α*‐cyano‐4‐hydroxy‐cinnamic acid (CHCA) and 2,5‐dihydroxybenzoic acid (DHB). The total ion count (TIC) for the standard mixture (including 10 ng mL^−1^ Ala, Arg, Glc, and Suc) showed 2–3 orders of magnitude enhancement using particles, in comparison with CHCA and DHB (Figure 1e). Subsequently, we evaluated the trapping performance for small metabolites in prepared solutions by mixing particles with the typical metabolite (Glc) and protein (BSA), respectively. We achieved molecular trapping ratios of 5.4 for Glc (typical small metabolite, *m*/*z* < 400 Da) and 2.5 for BSA (typical large molecule, *m*/*z* > 10,000 Da), by calculating the signal intensities of carbon element on the surface of particle‐molecule hybrids to the background in ten randomly selected area (5/5, surface/background, Figure 1f and Figure S3a,b and Table S3, Supporting Information), demonstrating the superior trapping performance for small metabolites. Further, to determine the minimum sample volume for MFFF, we evaluated the cosine similarity scores of spectra obtained from FF samples and their respective water dilutions at 2–100 folds (Figure 1g and Figure S3c, Supporting Information). The scores were all above 0.78 by utilizing 25–1000 nL of raw FF samples with up to 40‐fold dilution, attributed to the UV light absorptance at the wavelength of 355 nm and the laser energy transition efficiency of the particles (Figure S3d, Supporting Information). Therefore, we validated the high detection sensitivity of the PALDI‐MS with a LOD of ≈60 fmol in standard solution and the minimal consumption of 25 nL of FF for small metabolite detection.

For analytical reproducibility, we detected the standard solution containing three standard metabolites (including lysine [Lys], Glc, and Suc). The CVs using particles were found <3% for signal intensities at *m*/*z* values of 169.09 [Lys+Na]^+^, 203.05 [Glc+Na]^+^, and 365.11 [Suc+Na]^+^. Specifically, the CVs ranged from 2.4% to 2.9% (Figure 1h), indicating the desirable reproducibility of PALDI‐MS due to the uniform crystallization of particles with arithmetic mean height (Sa) of 0.251 µm (Figure S4a,b, Supporting Information). In contrast, the CVs were 50.2% to 387.3% using CHCA (Sa of 1.725 µm) and DHB (Sa of 7.471 µm) as the matrix (Figure S4c,d, Supporting Information). Further, we calculated the median CVs of *m*/*z* features extracted from FF samples. We obtained median CVs of 13.4% and 10.6% for normal ovarian reserve (nOR) and dOR samples, in five independent tests of each sample (Figure 1i). Thus, these results from both standard mixture (CVs of 2.4–2.9%) and real‐case biofluids (median CVs of 10.6–13.4%) indicated that our PALDI‐MS method would be a highly promising diagnostic tool for large‐scale clinical applications.

Attempts to profile metabolites using various methodologies focused primarily on two techniques, nuclear magnetic resonance (NMR) and MS. NMR, which was based on the electromagnetic properties of metabolites, required an acquisition time of ≈4–5 min and a large sample volume of approximately hundreds of microliters to elucidate the structure of metabolic components.^[^ ^16^ ^]^ Parallelly, for conventional MS, rigorous sample pretreatment (e.g., enrichment and deproteinization of ≈15 min) and large sample volume (≈10–400 uL) were required to exclude the presence of a high abundance of proteins and salts, thus reducing the effect of sample complexity.^[^ ^17^ ^]^ In this study, we applied ferric oxide particles as the matrix to enhance detection sensitivity and reproducibility. The homogeneous spatial distribution of matrix‐sample on the chip led to high reproducibility of mass signals, enhancing the analytical reproducibility for metabolite detection.^[^ ^14a^ ^]^ Thus, we demonstrated that PALDI‐MS allowed efficient MFFF with an analytical speed of ≈30 s per sample for the entire experiment by the microarray design and direct analysis of the FF samples. Further, considering the high sensitivity for metabolite detection (LOD of ≈60 fmol for standard solutions and minimal consumption of 25 nL biofluid) and desirable reproducibility (<15%), our platform for MFFF would serve as a powerful tool in encoding OR and fertility.

### 2.2 Characterization of OR and Fertility Using MFFF

**Figure 2:**
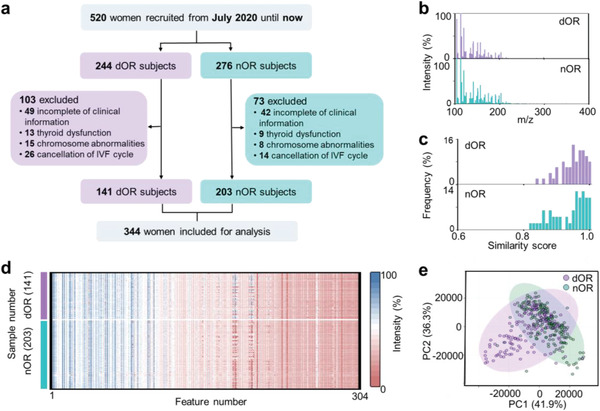
Characterization of OR and fertility using MFFF. a) Flowchart for the enrollment of subjects, highlighting the strict selection of 344 subjects out of 520 for inclusion in the study. Notably, the MFFF was performed on all 344 FF samples collected. b) Representative mass spectra of FF from dOR and nOR samples, showing TIC of ≈8.34–8.53 × 10^7^ at *m*/*z* of 100–400 Da. c) Frequency distribution of intragroup similarity scores of typical spectra from FF of the dOR group (50 samples) and nOR group (50 samples), indicating high levels of intra‐group similarity scores with 95% of FF samples having scores >0.85. d) Heatmap visualization of MFFF including 304 features from 141 dOR and 203 nOR samples. The FF samples were diluted at tenfolds using water. The color bars represented intensities corrected by logarithms. e) Unsupervised clustering by PCA revealing rough separation between dOR (cyan dots) and nOR (purple dots) groups.

In the case‐control design, FF samples were selected from a OR biobank with 520 subjects at the Reproductive Center of the First Affiliated Hospital of Anhui Medical University in China (**Figure** **2**a). Of these, we selected 344 FF samples including 141 dOR subjects and 203 nOR subjects. Notably, for the dOR group, we used a combination of test results, including FSH, AMH, AFC, the number of high‐quality oocytes (HQO), and the number of high‐quality embryos (HQE), to verify all subjects in this study (see Experimental Section for detail and Tables S4 and S5, Supporting Information). At the time of recruitment, we recorded the baseline information, including clinical parameters and medical history. For the nOR group, enrollment of participants was conducted based on the criteria as women 1) with complete clinical information, 2) without thyroid dysfunction, 3) without chromosome abnormalities, and 4) with no cancellation of the IVF cycle. All FF samples were collected on the day of oocyte retrieval to ensure the standard collection of the samples. Therefore, given the strict criteria for selecting dOR and nOR groups, the FF samples in this cohort would be representative of encoding OR and fertility.

Further, we constructed a metabolic database of FF for encoding OR and fertility based on PALDI‐MS. The mass spectra of FF samples were obtained with TIC of ≈8.34–8.53 × 10^7^ at the *m*/*z* range of 100–400 Da (Figure 2b). We collected ≈120, 000 datapoints from the raw mass spectrum of each FF sample due to the high resolution (0.005 Da) of PALDI‐MS. Notably, the typical mass spectra showed high similarity levels with more than 95% of FF samples having scores over 0.85 in each group (Figure 2c), which suggested its reliability and potential for diagnostic and predictive use. Moreover, the heatmap visualization of MFFF containing 304 *m*/*z* features was achieved by detecting and aligning peaks from the native data ranging from 100 to 400 Da, forming the blueprint of MFFF including 141 dOR and 203 nOR samples (Figure 2d). Through unsupervised clustering of principal component analysis (PCA) and t‐distributed stochastic neighbor embedding (t‐SNE), MFFF of OR samples showed rough separation between dOR and nOR samples (Figure 2e and Figure S5a, Supporting Information), implying the need to introduce advanced machine learning algorithms to differentiate dOR from nOR.

The success of the case‐control study was largely dependent on the sample size and data quality.^[^ ^18^ ^]^ For sample size, a total of 344 well‐defined subjects were enrolled in this study to encode OR using FF samples in Figure 2 and Tables S4 and S5, Supporting Information (see Experimental Section for details). A power analysis of ten samples (5/5, dOR/nOR) was conducted as a preliminary study to determine the minimum sample size that would allow us to perform machine learning significantly. With 100 samples per group (100/100, dOR/nOR) and a false discovery rate (FDR) of 0.10 (Figure S5b, Supporting Information), we can obtain a predictive power of >0.82, indicating the reliability of the subsequent machine learning results.

For data quality, sample selection and data acquisition were crucial. As the fluid surrounding the oocyte within the ovary, FF composition provided insights into physiological signaling processes and served as a real‐time indicator of the ovarian state, demonstrating its potential as a predictor of OR and fertility.^[^ ^19^ ^]^ Furthermore, FF was the byproduct of oocyte retrieval, allowing for acquisition in a non‐invasive manner.^[^ ^20^ ^]^ For data acquisition, we received ≈124, 000 datapoints at a mass resolution of 0.005 Da and extracted 304 *m*/*z* features for each sample. Moreover, more than 95% of FF samples showed similarity scores higher than 0.85 of intra‐groups, demonstrating the high quality and consistency of the metabolic data. Therefore, we constructed a robust database recording metabolic information in FF from 344 subjects for the following metabolic analysis of FF.

### 2.3 Machine Learning of MFFF for dOR Diagnosis and Fertility Prediction

**Figure 3:**
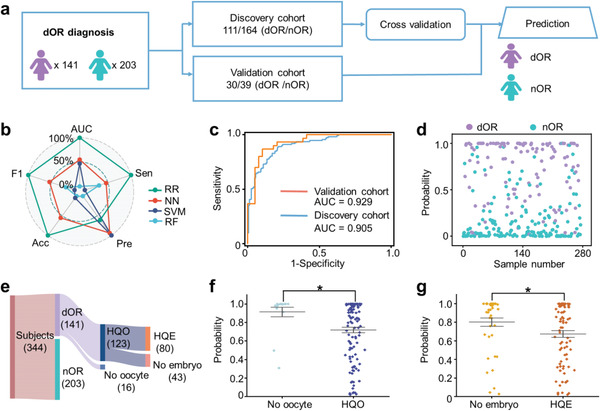
Encoding of OR and fertility by machine learning of MFFF. a) A study design based on machine learning to diagnose dOR. There were 275 FF samples (111/164, dOR/nOR) included in the discovery cohort for cross‐validation. An independent validation cohort was used to assess the optimized model (30/39, dOR/nOR). b) The model performance of RR, NN, SVM, and RF for dOR diagnosis in terms of AUC, Sen, Pre, Acc, and F1. A min‐max normalization was used to normalize these parameters. c) The ROC curves of dOR diagnosis in the discovery cohort (111/164, dOR/nOR, blue line) and the independent validation cohort (30/39, dOR/nOR, orange line). d) The sample‐level plot depicting the probability of RR in the discovery cohort for differentiating dOR (111, purple dots) from nOR (164, cyan dots). e) Fertility information (the number of subjects with HQO and HQE) of the enrolled subjects (141/203, dOR/nOR). f) The probability of the HQO group and no oocyte group, showing a significant difference in predicting oocyte quality (*p* < 0.05). The *p‐*value was calculated by a two‐tailed *t*‐test. * represented *p* < 0.05. g) The probability of HQE group and no embryo group, displaying a significant difference in predicting embryo quality (*p* < 0.05). The *p‐*value was calculated by a two‐tailed *t*‐test. * represented *p* < 0.05.

We performed machine learning of MFFF for encoding OR and fertility. A total of 275 subjects (111/164, dOR/nOR) were enrolled and randomly allocated to the discovery cohort for tenfold cross‐validation (**Figure** **3**a). The age in the discovery cohort was matched with no significant difference between the dOR and nOR groups (*p* > 0.05, Table S6, Supporting Information). The independent validation cohort was constructed with the remaining 69 subjects (30/39, dOR/nOR). Subsequently, we conducted model building using four algorithms (ridge regression [RR], neural network [NN], support vector machine [SVM], and random forest [RF]) for dOR diagnosis. Analysis of the model performance was carried out in terms of AUC, accuracy (Acc), sensitivity (Sen), precision (Pre), and F1 score (F1, harmonic mean of Sen and Pre, Figure 3b and Table S7, Supporting Information). All four algorithms achieved AUC ≥ 0.79, indicating the diagnostic potential of MFFF for dOR. Importantly, RR had a significantly better AUC of 0.905 with a 95% confidence interval (CI) of 0.870–0.940 in comparison to NN (AUC of 0.856 with 95% CI of 0.812–0.900), SVM (AUC of 0.847 with 95% CI of 0.801–0.893), and RF (AUC of 0.796 with 95% CI of 0.744–0.849) in the discovery cohort (*p* < 0.05 by Delong test, Figure 3b). Our results were consistently replicated in the independent validation cohort with an AUC of 0.929 (95% CI of 0.867–0.991, Figure 3c and Table S8, Supporting Information). Moreover, we performed a permutation test yielding *p* < 0.0002 (Figure S5c, Supporting Information), demonstrating the efficacy of the RR model in dOR diagnosis without overfitting. Further, we generated sample‐level plots to depict the predictive probability for differentiating dOR samples from nOR samples (Figure 3d and Figure S5d, Supporting Information). The above results from the RR algorithm in the discovery cohort (AUC of 0.905) and independent validation cohort (AUC of 0.929) demonstrated the diagnostic capacity of machine learning using MFFF for dOR diagnosis.

To further investigate the predictive capability of MFFF for fertility, we classified the dOR subjects who underwent IVF into the HQO group and HQE group according to fertility information. There were 139 subjects (123/16, HQO group/no oocyte group, 80/43, HQE group/no embryo group) after excluding the subjects without oocyte information (Figure 3e). Scatter plot analysis revealed a statistically significant difference in the average probabilities between the HQO and no oocyte groups (*p* < 0.05, Figure 3f). In parallel, FSH levels exhibited a statistically significant difference (*p* < 0.05, Figure S6a, Supporting Information), while AFC levels displayed no significant difference (Figure S6b, Supporting Information). Furthermore, for the prediction of HQE, the HQE group exhibited a lower probability than the group with no embryo (*p* < 0.05, Figure 3g), demonstrating the predictive capability of MFFF for embryo quality. In contrast, the FSH and AFC both showed no significant difference between the HQE group and the no embryo group (Figure S6c,d, Supporting Information). Therefore, we constructed a robust multi‐functional platform for dOR diagnosis (AUC of 0.905–0.929) and fertility prediction (determining HQO and HQE with *p* < 0.05) using machine learning of MFFF, thus providing an efficient tool for encoding OR and fertility toward clinical applications.

Accurate encoding of the disease was essential for choosing the treatment strategies and slowing down the disease's progression.^[^ ^14a^ ^]^ Importantly, there is no single test yet to encode OR and fertility, covering DOR diagnosis and HQO/HQE determination. Current analytical methods for encoding OR and fertility, requiring a combination of multiple tests (e.g., FSH, AMH, and AFC), are limited for clinical applications due to the inherent features lacking enough information from the specific biofluids in the ovary. Specifically, with an AUC of <0.70 for OR encoding, the diagnostic performance of FSH limited their reliability owing to intracycle or intercycle variability of FSH.^[^ ^21^ ^]^ Considering that AMH was circulating in the blood and lacks an international test standard, the diagnostic AUC of AMH was limited to 0.63–0.78, and thus it is challenging to encode OR using the sole biomarker of AMH.^[^ ^4^, ^22^ ^]^ In parallel, the examination of AFC needed to count the primary medial follicles under ultrasound on the day of 2–5 of the menstrual cycle by experienced doctors, affording AUC < 0.75.^[^ ^23^ ^]^ Moreover, for the prediction of fertility, predictors using sole biomarkers (e.g., AFC and FSH) showed poor prediction performance for HQO and HQE with no significant difference (*p* > 0.05, Figure S6, Supporting Information).

For comparison, the MFFF realized efficient encoding of OR and fertility by a single test (Figure 3). The machine learning of MFFF showed superior results (AUC of 0.929) for dOR diagnosis than previous studies using conventional and metabolic biomarkers (AUC of ≈0.63–0.85), owing to the advanced interpreting of MFFF by machine learning.^[^ ^4^, ^9^, ^24^ ^]^ Notably, our method also showed predictive potential to determine HQO and HQE (*p* < 0.05) with a single test. Therefore, our platform would be a multi‐functional platform that effectively utilized MFFF to encode OR and fertility, providing an effective alternative to existing tools in clinical settings.

### 2.4 Biomarker Panel Construction and Related Pathway Analysis

**Figure 4:**
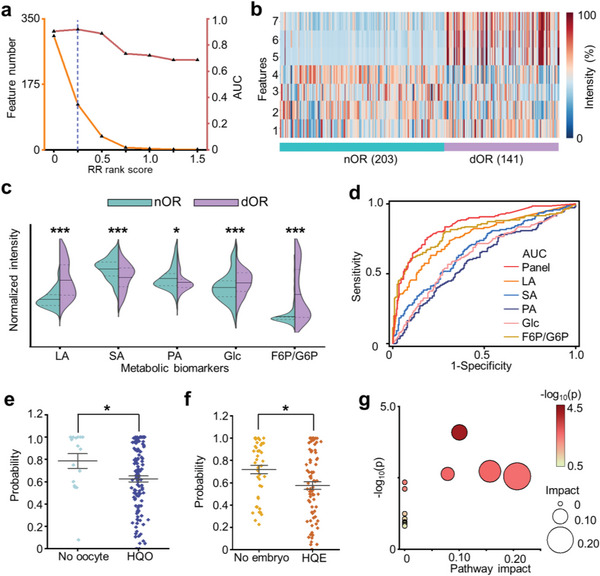
Biomarker panel construction and related pathway analysis. a) Feature number (orange line) and AUC of RR (red line) calculated at various thresholds of RR rank score in the discovery cohort. Blue dotted lines indicated the optimized AUC of 0.919 at the threshold of 0.25. b) Intensity heatmap of 7 *m*/*z* features showing distinct expression levels between dOR and nOR. The color bars represented intensities corrected by logarithms. c) The violin plot displaying the intensity levels of identified biomarkers for dOR and nOR. A two‐tailed *t*‐test was used to determine the *p* values (* represented *p* < 0.05, ** represented *p* < 0.005, *** represented *p* < 0.001). The biomarker intensities were normalized. d) The ROC curves for the biomarker panel with an AUC of 0.849, superior to the single biomarker with an AUC of 0.571–0.803 (*p* < 0.05 by Delong test). e) The comparison of probability for the HQO group and no oocyte group in predicting oocyte quality using constructed biomarker panel (* represented *p* < 0.05). A two‐tailed *t*‐test was conducted to calculate the *p* values. f) The comparison of probability for the HQE group and no embryo group in predicting embryo quality using constructed biomarker panel (* represented *p* < 0.05). A two‐tailed *t*‐test was conducted to calculate the *p* values. g) The potential pathways associated with dOR and nOR. The size and color of the circles represented the p‐value and pathway impact, respectively.

We developed a metabolic biomarker panel through feature selection and identified related metabolic pathways, toward clinical application for encoding OR and fertility. Specifically, we optimized the AUC of the RR model by setting various thresholds of RR rank score in the discovery cohort (**Figure** **4**a). The AUC reached 0.919 with an RR rank score of 0.25, higher than the AUC values (0.686–0.905) in other thresholds. There were 121 *m*/*z* features selected from MFFF with the optimized AUC, from which 7 *m*/*z* features were selected as promising candidates for biomarker identification using the criteria of mean intensity ($\bar{I}$) > 3000 and *p* < 0.05. The unsupervised clustering analysis by the selected 7 *m*/*z* features showed a distinct intensity level between dOR and nOR (Figure 4b), demonstrating the capability of these features for dOR diagnosis.

Subsequently, we identified lactic acid (LA), succinic acid (SA), pyruvic acid (PA), Glc, and fructose 6‐phosphate/glucose 6‐phosphate (F6P/G6P) as the biomarker panel from the selected *m*/*z* features. The biomarkers were further validated using Fourier‐transform ion‐cyclotron resonance mass spectrometry (FT‐ICR MS, Table S9, Supporting Information) and LC‐MS (Table S10, Supporting Information). Compared to nOR group, the LA, Glc, and F6P/G6P were over‐expressed (*p* < 0.001), while SA and PA were down‐expressed (*p* < 0.05) in the dOR group (Figure 3c). The constructed biomarker panel showed promising diagnostic performance with an AUC of 0.849 (95% CI: 0.809‐0.890), superior to the single biomarker with an AUC of 0.571–0.803 (Figure 4d and Table S11, Supporting Information). To further evaluate the biomarker panel for predicting fertility, we calculated the probability of the machine learning model trained by the biomarker panel for differentiating HQO and HQE. We found a significant difference in probability between HQO and no oocyte groups (*p* < 0.05, Figure 4e). The probability of HQE and no embryos groups also exhibited significant differences as well (*p* < 0.05, Figure 4f). Therefore, we validated the constructed biomarker panel as an indicator for encoding OR (AUC of 0.849) and fertility (*p* < 0.05 for determining HQO and HQE).

We also conducted metabolic pathway analysis correlated to the constructed biomarker panel. Four pathways related to OR and fertility were screened with pathway impact (PI) > 0, including 1) pyruvate metabolism (PI of 0.21), 2) starch and sucrose metabolism (PI of 0.14), 3) glycolysis/gluconeogenesis (PI of 0.10), and 4) citrate cycle (PI of 0.08, Figure 4g). Specifically, in pyruvate metabolism, the abnormal expression of LA and PA affects the quality and maturation of the oocyte, thus elevating the risk of dOR and infertility.^[^ ^25^ ^]^ For starch and sucrose metabolism, the alternations in G6P levels have the potential to disrupt the integrity of DNA in the oocyte through the nucleotide production process. In the pathway of glycolysis/gluconeogenesis, the Glc and PA are of paramount importance in facilitating energy supply for oocyte development and maturation, and any dysfunction in this pathway could impact ovarian function.^[^ ^26^ ^]^ Moreover, high levels of LA may impair follicle growth and maturation, leading to dOR and a reduced number of oocytes.^[^ ^27^ ^]^ In the citrate cycle, the altered SA and PA could reduce adenosine triphosphate (ATP) production, thereby negatively impacting oocyte development, maturation, and ovulation.^[^ ^28^ ^]^ The above pathway analysis using metabolites as the basis can help to provide a comprehensive understanding of OR and fertility and insights into the reproductive potential before transplantation.

Molecular biomarkers, including nucleic acids, proteins, and metabolites in specific biofluids, were increasingly used in diagnostic applications, due to their accuracy and convenience for clinical use.^[^ ^29^ ^]^ Conventionally, reports relying on the upstream biomarkers of nucleic acids and proteins showed diagnostic AUC of 0.63–0.82 for DOR diagnosis based on signal amplification or biochemical reaction, which owned low analytical speed of minutes to hours and sample volume of ≈30–100 µL.^[^ ^5^, ^22^, ^30^ ^]^ In parallel, the analyses using downstream metabolic biomarkers for OR and fertility were still preliminary, owing to the limited number of subjects (≈40–150) and lack of multi‐functional tools, especially for specific biofluid of FF.^[^ ^9^, ^10^ ^]^ In our work, we constructed a biomarker panel for encoding OR and fertility using metabolites in FF from a cohort of 344 subjects, providing satisfactory performance with an AUC of 0.849 in a label‐free manner. Moreover, our platform based on PALDI‐MS allowed MFFF with fast analytical speed (≈30 s) and trace sample volume (down to 25 nL). Therefore, we built an efficient platform to detect FF metabolites with multi‐function, toward rapid and accurate encoding OR and fertility.

## 3 Conclusion

For the future perspective of this work, the performance of metabolic biomarkers would be further enhanced by incorporating additional biomarkers into a multi‐modal database. Moreover, biological validations could be conducted to understand the underlying mechanism and could lead to the development of new therapeutic strategies to improve fertility outcomes.

In summary, we performed efficient MFFF using PALDI‐MS and constructed a biomarker panel for encoding OR and fertility. We achieved efficient MFFF with fast speed (≈30 s), high sensitivity (≈60 fmol), and desirable reproducibility (CVs < 15%) by PALDI‐MS. Further, we applied machine learning of MFFF to diagnose dOR with an AUC of 0.929 and identify high‐quality oocytes/embryos (*p* < 0.05) by a single PALDI‐MS test. Subsequently, we constructed a biomarker panel showing an AUC of 0.849 for dOR diagnosis and effective determination of high‐quality oocytes/embryos (*p* < 0.05). Our work would provide a powerful tool for women's healthcare including but not limited to OR and fertility.

## 4 Experimental Section

### Chemicals and Reagents

In this study, the chemicals and reagents included: 1) matrix materials, 2) standard metabolites, and 3) other reagents and chemicals.

For the matrix materials, the particles were prepared using high‐purity iron chloride hexahydrate (FeCl_3_·6H_2_O, 99%), trisodium citrate dihydrate (99.5%), sodium acetate anhydrous (99%), ethylene glycol (99.5%), and ethanol absolute (99.7%), all of which were procured from Sinopharm Chemical Reagent Beijing Co., Ltd. (Beijing, China). The organic matrices of CHCA and DHB were purchased from Sigma‐Aldrich (St. Louis, MO, USA).

The standard metabolites of amino acids and sugars, including Ala (98%), Val (98%), Lys (98%), Arg (98%), Glc (99.5%), and Suc (99%), were ordered from Sigma‐Aldrich (St. Louis, MO, USA).

For other reagents and chemicals, sodium chloride (NaCl, 99.5%) and BSA (98%) were purchased from Sigma‐Aldrich (St. Louis, MO, USA). Acetonitrile (ACN, 99%) was procured from Aladdin Reagent (Shanghai, China). Methanol (HPLC) was obtained from ThermoFisher Scientific (Waltham, USA). Trifluoroacetic acid (TFA, 99.5%) was purchased from Macklin Biochemical Co., Ltd. (Shanghai, China). All experiments were conducted using purified water (18.2 MΩ cm) generated by a Millipore Milli‐Q system (Milli‐Q, Millipore, GmbH).

### Preparation of Particles

A hydrothermal method was used for the large‐scale synthesis of particles according to previous work.^[^ ^5^, ^12^, ^14^ ^]^ Specifically, trisodium citrate dihydrate and iron chloride hexahydrate were first dissolved in ethylene glycol with shaking. Then sodium acetate was added to the above mixture under ultrasonic vibration treatment. The resulting mixture was then placed in the autoclave for hydrothermal treatment at 200 °C. After a 10 h reaction, the solid particles were washed using ethanol absolute and purified water. Finally, the obtained particles were dried at 60 °C and saved for later use.

### Characterization of the Particles

Transmission electron microscopy (TEM), high‐resolution TEM (HRTEM), and elemental mapping analysis were conducted using JEOL JEM‐2100F (JEOL Ltd., Japan) by dispensing 3 µL aqueous solution of particles or molecule‐particle hybrids onto a copper grid. The scanning electron microscopy (SEM) images were collected using S‐4800 (Hitachi Ltd., Japan) by depositing 1.5 µL of the aqueous solution of particles on a silicon wafer. The bright‐field images for the characterization of crystallization were captured by Eclipse Ti (Nikon Ltd., Japan). The confocal scanning images were performed using VK‐X3000 (KEYENCE Ltd., Japan). The microarray was imaged using a P40 Pro smartphone (Huawei Technologies Co., Ltd., China).

### Study Population

This work designed a case‐control study of women who underwent at least one fresh cycle of IVF or intracytoplasmic sperm injection (ICSI) at the Reproductive Center of the First Affiliated Hospital of Anhui Medical University in China from July 2020 until now. Case groups were defined as follows: 1) The year of non‐pregnant was more than 1 year; 2) at least two of the following criteria were met for dOR diagnosis: FSH was greater than 10 IU L^−1^; AFC was less than 5; AMH was lower than 1.1 ng mL^−1^. Patients with any of the following conditions were excluded from the control group: 1) incomplete clinical information; 2) thyroid dysfunction; 3) chromosome abnormalities; 4) cancellation of IVF cycle.

A total of 344 FF samples from 520 subjects aged 20–50 years were collected in this study. Among the 344 samples, there were 203 dOR samples and 141 dOR samples. After excluding the subjects without oocyte information, there were 123 subjects with HQO and 16 subjects without oocytes. Of the 123 patients who underwent IVF, 80 obtained HQE, and 43 failed to get embryos. The study was approved by the Ethical Committee of Anhui Medical University (PJ2020‐07‐22). Oral and written consent was obtained from all subjects. The study complied with the Declaration of Helsinki and involved medical research with human subjects.

### Collection of FF Samples

FF was collected on the day of oocyte retrieval.^[^ ^20^ ^]^ The FF from the largest follicle was meticulously collected into a 15 mL centrifuge tube. The collected FF sample was centrifugated at a rate of 2000 revolutions per minute for 15 min, after which the resulting supernatant was carefully segregated into 1 mL aliquots. Then, the supernatant was stored at an ultra‐low temperature of −80 °C until further analysis.

### Statistical Analysis

For the age match of dOR and nOR subjects, a two‐tailed *t*‐test was conducted using Microsoft Excel (version 2021). No statistically significant difference was found between the dOR and nOR groups in the discovery cohort. The similarity scores and Delong test were conducted through MATLAB (version R2022a, The Math Works, USA) using homemade code.

For the determination of the minimum sample size in machine learning, a power analysis was conducted on the website of MetaboAnalyst 5.0 (https://www.metaboanalyst.ca/). The MFFF of ten samples (5/5, dOR/ nOR) was randomly chosen for the power analysis to calculate the significant sample size with an FDR of 0.1. The significant levels for *p*‐values were set to 0.05.

### Metabolic Analysis of Standard Metabolites

The organic matrices of CHCA and DHB were dispersed in a solution of TA30 consisting of ACN and water (3:7, v/v) with 0.1% TFA, at a concentration of 10 mg mL^−1^. For matrix preparation, 1 mg mL^−1^ particle solution was obtained by dispersing the particles in water. The aqueous solutions were prepared for standard metabolite detection by dissolving small molecules in water with 1 mg mL^−1^. In a typical detection of salt and protein tolerance, the standard metabolites, including Val, Lys, Arg, and Glc, were mixed with 0.5 m NaCl or 5 mg mL^−1^ BSA for MS analysis.

To determine LOD, the typical metabolites (Glc, Suc, Ala, Arg) at the concentration from 5E‐05 to 1 mg mL^−1^ were detected.^[^ ^31^ ^]^ Then the linear regression analysis of intensities and related concentration data was performed to acquire the regression equation. The calibration curves were obtained for typical metabolites using particles, CHCA, and DHB as the matrix. The LOD of the particle was calculated using the slope of the calibration curve and the signal‐to‐noise ratio (S/N) of 3. The LOD of CHCA and DHB were calculated using metabolite peaks with S/N > 3, due to the R^2^ values of regression equation for CHCA and DHB being < 0.8.

### Metabolic Analysis of FF Samples

The FF samples were detected directly with minimum sample pretreatment. Specifically, the FF samples were diluted at tenfolds using water after the pretreatment optimization. In a typical process of metabolic fingerprinting, 1 µL of diluted FF sample was deposited onto a microarray chip. Then 1 µL of the particle solution with a concentration of 1 mg mL^−1^ was distributed on the dried FF sample.

The mass spectra of metabolites based on PALDI‐MS were recorded by Bruker Autoflex MALDI TOF/TOF mass spectrometer (Brucker Daltonics, Bremen, Germany) in the positive ion mode, with Nd:YAG lasers of 355 nm and a laser moving diameter of 2000 um.^[^ ^32^ ^]^ During the detection process, the pulse laser was optimized at a frequency of 1000 Hz with 2000 shots. The instrument's delay time and acceleration voltage were 150 ns and 120 kV, respectively. Further, the biomarker was identified through the Solarix 7.0T (FT‐ICR MS, Brucker Daltonics, Germany) and LC‐MS analysis using Q Exactive plus (ThermoFisher Scientific, USA).

For conducting FT‐ICR MS, the collected FF samples were diluted using water at tenfolds. Then, 1 µL of the diluted FF samples were dropped on the micro‐array chip to dry at room temperature. The matrix solution of the particle was dropped on the dried sample for the following FT‐ICR MS analysis.

For conducting LC‐MS, the FF samples were pretreated using the following steps: protein precipitation, supernatant collection, and reconstitution. 200 µL of organic solvent (methanol/ACN, 1/1, v/v) was added to 50 µL of collected FF samples, followed by placing the mixture in a refrigerator at −20 °C for 2 h to precipitate the proteins. Subsequently, the mixture was centrifuged at 12 000 rpm for 15 min. Then the supernatant of the mixture was transferred into a clean microcentrifuge tube and dried using a vacuum concentrator. The dried samples were reconstituted in a methanol solution (methanol/water, 3/7, v/v), vortexed for 3 min, and centrifuged at 12 000 rpm for 15 min to remove insoluble components. Finally, the supernatant was collected for the following LC‐MS analysis.

The pathway analysis was conducted on MetaboAnalyst 5.0 (https://www.metaboanalyst.ca/).^[^ ^33^ ^]^ Typically, the metabolite sets, including selected biomarkers, were analyzed according to the library of Kyoto Encyclopedia of Genes and Genomes (KEGG) database using a hypergeometric test on MetaboAnalyst 5.0.

### Machine Learning of MFFF

To facilitate the machine learning of MFFF, unsupervised analysis was initially employed to assess the discriminative performance between dOR and nOR. The unsupervised analysis of PCA and t‐SNE was utilized to visualize the MFFF data of all FF samples.

For the dataset dividing, the dataset was divided into the discovery cohort and validation cohort in a random manner, with a ratio of ≈4:1 (275 subjects/69 subjects, discovery cohort/validation cohort) for all clinical subjects.

Subsequently, to evaluate the dOR diagnostic performance of MFFF, four machine learning algorithms, including RR, NN, SVM, and RF, were utilized for the model building in the discovery cohort and validation cohort. For parameters choosing, to select the optimal parameters, the machine learning models were trained on the 304 features of each FF sample and evaluated characterization performance in terms of AUC, Sen, Pre, Acc, and F1 score. Then, the predicted probability of dOR for each machine learning model was computed and measured AUC using ROC of the average predicted probability and the real label. The Sen, Pre, Acc, and F1 score were determined by comparing the predicted labels averaged across all samples with the actual labels by a probability threshold of 0.5. Machine learning of the MFFF was performed by the software of Orange (version 3.33.0, Slovenia).

### Biomarker Panel Construction

To construct the biomarker panel, multiple selection parameters were utilized including the RR rank score, *p‐*value, and mean intensities of each *m*/*z*. Typically, the performance of the RR model was optimized by setting various thresholds of RR rank score in the discovery cohort. The RR score was ultimately set as 0.25 to achieve the optimal AUC. Subsequently, the *m*/*z* features were selected according to the following criteria: 1) RR rank score > 0.25, 2) mean intensity ($\bar{I}$) > 3000, and 3) *p* < 0.05. The selected features were identified using LC‐MS, FT‐ICR MS, and database of human metabolome database (HMDB, https://hmdb.ca/) for the biomarker panel construction.

## Conflict of Interest

The authors declare competing financial interest. Both the technology and the method of detecting bio‐samples are patented by the authors.

## Author Contributions

Jia. Wu, C.L., and Xin Wang contributed equally to this work. K.Q., D.J., and Y.C. designed the conception of this work with Jia. Wu, C.L., and Xin Wang. C.L, Xin Wang, X.S., T.Y., Xia. Wang, Z.Z., L.S., D.L., W.Z., Ji Wu, L.Q., and W.D. contributed to the collection of FF samples. Jia. Wu conducted the MS experiments and prepared the particles. Jia. Wu, Y.H., W.L., R.W., and J.C. contributed to the data analysis. Jia. Wu wrote the manuscript with C.L. and Xin Wang. All authors joined in the critical discussion and edited the manuscript.

## Supporting information

Supporting Information

## Data Availability

The data that support the findings of this study are available from the corresponding author upon reasonable request.
