# Supplementary material for: Efficient Metabolic Fingerprinting of Follicular Fluid Encodes Ovarian Reserve and Fertility
Source: Adv Sci (Weinh). 2023 Jun 13;10(23):2302023. doi: 10.1002/advs.202302023 (PMC10427401; doi:10.1002/advs.202302023)
Supplement: Supplementary file 1 — Supporting Information [file ADVS-10-2302023-s001.pdf]

ADVANCED SCIENCE
Open Access

Supporting Information

for *Adv. Sci.*, DOI 10.1002/advs.202302023

Efficient Metabolic Fingerprinting of Follicular Fluid Encodes Ovarian Reserve and Fertility

Jiao Wu, Chunmei Liang, Xin Wang, Yida Huang, Wanshan Liu, Ruimin Wang, Jing Cao, Xun Su, Tao Yin, Xiaolei Wang, Zhikang Zhang, Lingchao Shen, Danyang Li, Weiwei Zou, Ji Wu, Lihua Qiu, Wen Di, Yunxia Cao, Dongmei Ji* and Kun Qian**

Efficient metabolic fingerprinting of follicular fluid encodes ovarian reserve and fertility

Jiao Wu[1,2,3†], Chunmei Liang[4,5,6†], Xin Wang[4†], Yida Huang[1,2,3], Wanshan Liu[1,2,3], Ruimin Wang[1,2,3], Jing Cao[1,2,3], Xun Su[4], Tao Yin[4], Xiaolei Wang[4], Zhikang Zhang[4], Lingchao Shen[4], Danyang Li[4], Weiwei Zou[4,5,6], Ji Wu[7,8], Lihua Qiu,[1,9] Wen Di,[1,9] Yunxia Cao[4,5,6]*, Dongmei Ji[4,5,6]*, Kun Qian[1,2,3]*

Affiliation

[1]Shanghai Key Laboratory of Gynecologic Oncology, Department of Obstetrics and Gynecology, Renji Hospital, School of Medicine, Shanghai Jiao Tong University, Shanghai 200127, P. R. China

[2]State Key Laboratory for Oncogenes and Related Genes, School of Biomedical Engineering and Institute of Medical Robotics, Shanghai Jiao Tong University, Shanghai 200030, P. R. China

[3]Division of Cardiology, Renji Hospital, School of Medicine, Shanghai Jiao Tong University, Shanghai 200127, P. R. China

[4]Department of Obstetrics and Gynecology, the First Affiliated Hospital of Anhui Medical University, Hefei 230022, P. R. China.

[5]NHC Key Laboratory of Study on Abnormal Gametes and Reproductive Tract, Anhui Medical University, Hefei 230032, P. R. China.

[6]Key Laboratory of Population Health Across Life Cycle, Anhui Medical University, Hefei 230032, P. R China.

[7]Key Laboratory for the Genetics of Developmental & Neuropsychiatric Disorders (Ministry of Education), Renji Hospital, Bio-X Institutes, School of Medicine, Shanghai Jiao Tong University, Shanghai 200240, P. R China.

[8]Key Laboratory of Fertility Preservation and Maintenance of Ministry of Education, Ningxia Medical University, Yinchuan 750003, P. R China.

[9]Department of Obstetrics and Gynecology, Ren Ji Hospital, School of Medicine, Shanghai Jiao Tong University, Shanghai 200127, P. R. China

†Jiao Wu, Chunmei Liang, Xin Wang contributed equally to this work.

*Corresponding authors. Email: k.qian@sjtu.edu.cn, jidongmei@ahmu.edu.cn, caoyunxia5972@ahmu.edu.cn

Contents

Supplementary Figures (1-6)
Supplementary Tables (1-11)

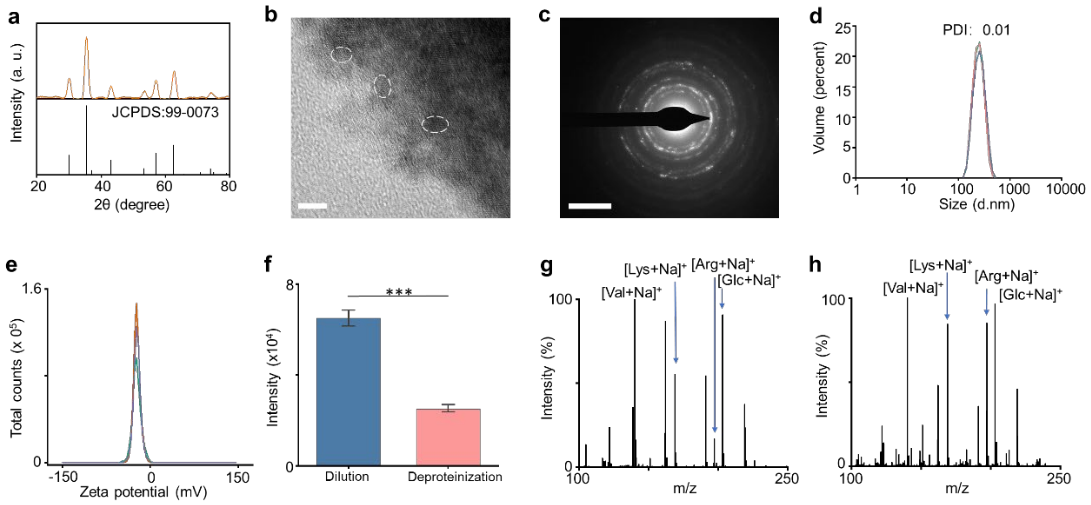

Figure S1 Characterization of the particles and detection of metabolites. a) X-ray diffraction patterns of particles with Joint Committee on Powder Diffraction Standards (JCPDS):99-0073 corresponding to Fe3O4. b) High resolution transmission electron microscopy (HRTEM) image of the edge region of particle, showing crystal structure as indicated by the white circles. The scale bar represented 5 nm. c) Selected area electron diffraction (SAED) pattern displaying the polycrystalline structure of the particle. The scale bar was $5nm^{-1}$. d) Dynamic light scattering of particles showing the uniform size with polydispersity index of 0.01 by 3 independent measurements. e) The zeta potential of particles displaying a negative charge of the material surface. f) The intensities of typical metabolite (Glucose, Glc) detected by particle assisted laser desorption/ionization mass spectrometry (PALDI-MS) after dilution and deproteinization treatments. The scale bars represented ± s.d. of 5 replicates. *** represented $p < 0.001$. Representative mass spectra of 1mg/ml Glc, arginine (Arg), lysine (Lys), and valine (Val) in g) 0.5 mol/L NaCl and h) 5 mg/mL BSA solutions.

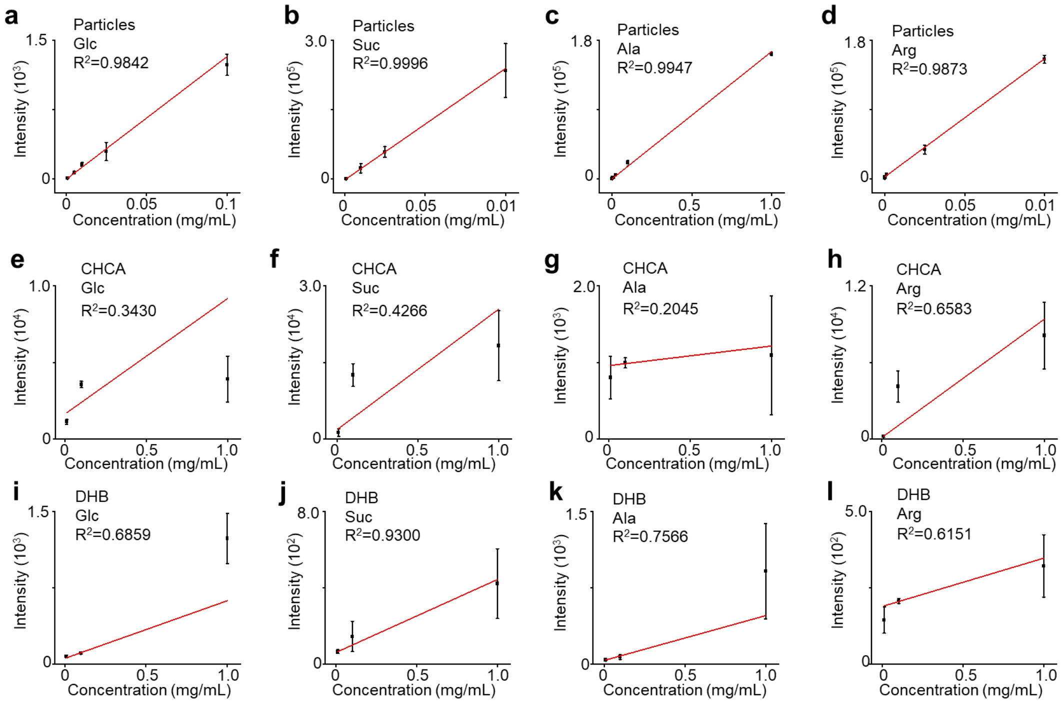

Figure S2 The calibration curves for detecting typical metabolites. The calibration curves for detecting a) Glc, b) Suc, c) Ala, and d) Arg using particles as the matrix. The calibration curves for detecting e) Glc, f) Suc, g) Ala, and h) Arg using CHCA as the matrix. The calibration curves for detecting i) Glc, j) Suc, k) Ala, and l) Arg using DHB as the matrix.

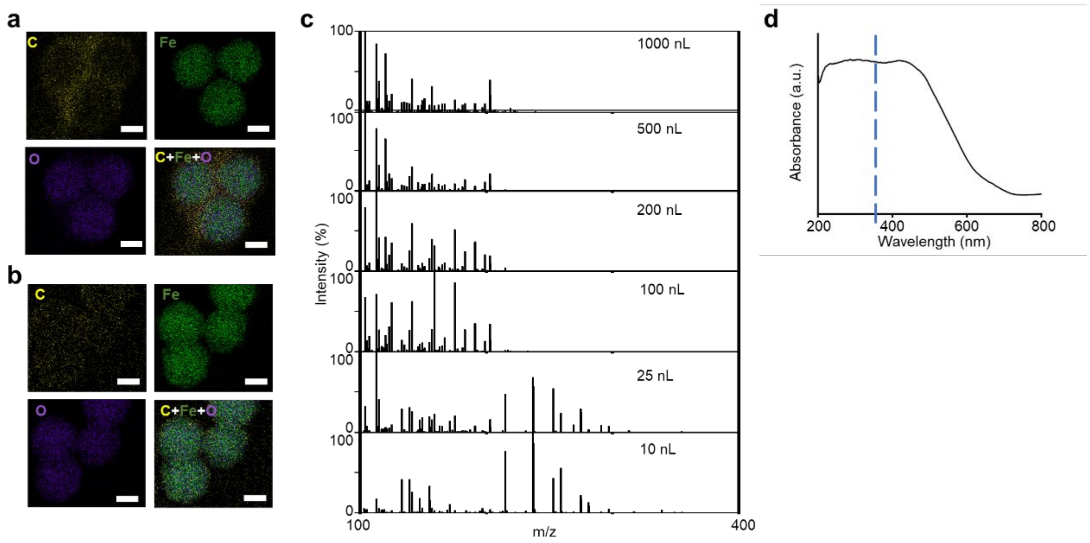

Figure S3 Characterization of PALDI-MS for sensitive detection. The elemental mapping results of a) particle-Glc and b) particle-BSA hybrids with carbon (C) element in yellow, ferrum (Fe) element in green, and oxygen (O) element in purple, showing the trapping performance for molecules. The scale bars represented 100 nm. c) The mass spectra of 1000 nL of raw follicular fluid (FF) sample and its dilutions by 2-100 folds using 10-500 nL of raw FF samples at the m/z ranging from 100 to 400 Da. d) The ultraviolet absorption spectrum of particles displaying the absorbance at 355 nm indicated by the blue dotted line.

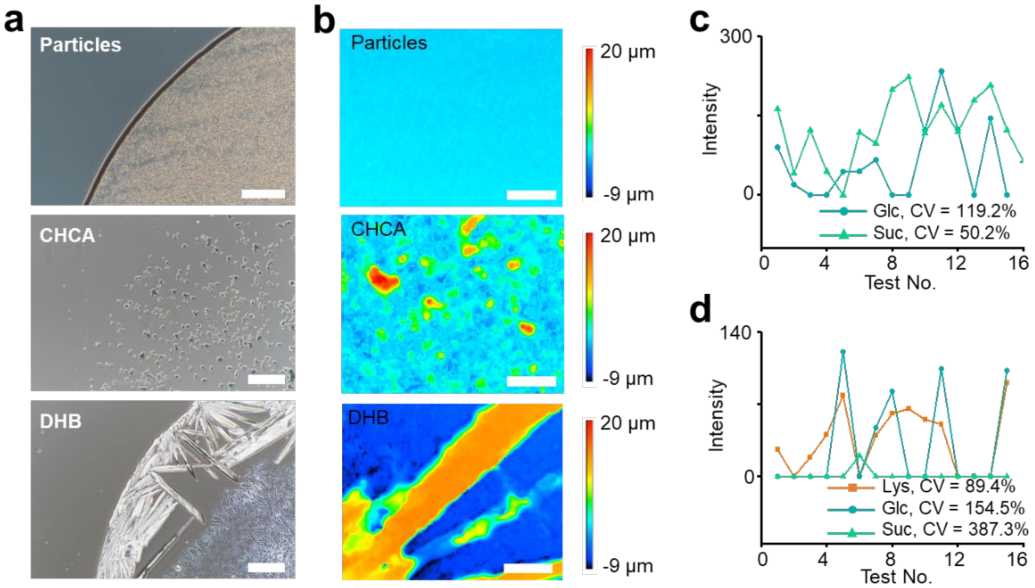

Figure S4 Characterization of the matrices and the CVs of standard solutions. a) The bright field images of particles, CHCA and DHB showing the crystallization of matrix. The scale bars of particles, CHCA, and DHB were 100 μm, 200 μm, and 200 μm, respectively. b) The confocal scanning images of matrix-sample co-crystallization using particles, CHCA, and DHB. The scale bar represented 50 μm. The intensities and coefficients of variations (CVs) of intensities for a mixture including lysine (Lys), Glc, and Suc in 15 tests using c) CHCA and d) DHB as the matrix. The Lys was undetectable using CHCA as the matrix.

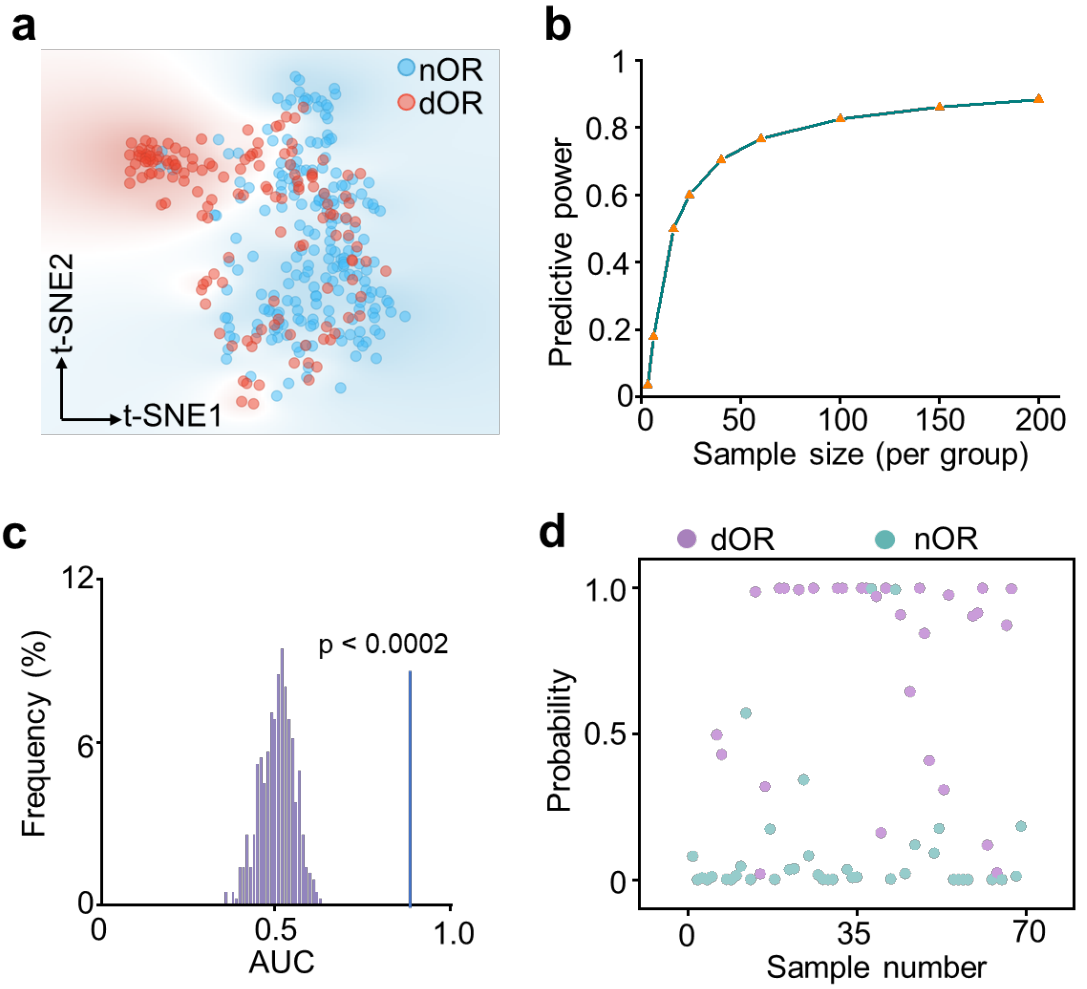

Figure S5 Characterization of MFFF. a) The t-distributed stochastic neighbor embedding (t-SNE) of metabolic fingerprinting of FF (MFFF), showing a certain degree of separation for diminished ovarian reserve (dOR) and normal ovarian reserve (nOR) samples. b) The power analysis using MFFF of 10 samples (5/5, dOR/nOR) to calculate the minimum sample size of each group with statistical significance for machine learning. c) The frequency distribution of AUC calculated by 5000 random permutations of RR, showing no overfitting of the model with a significant p-value of < 0.0002. d) The sample-level plot depicting the probability of RR in the independent validation cohort for differentiating dOR (30, purple dots) from nOR (39, cyan dots).

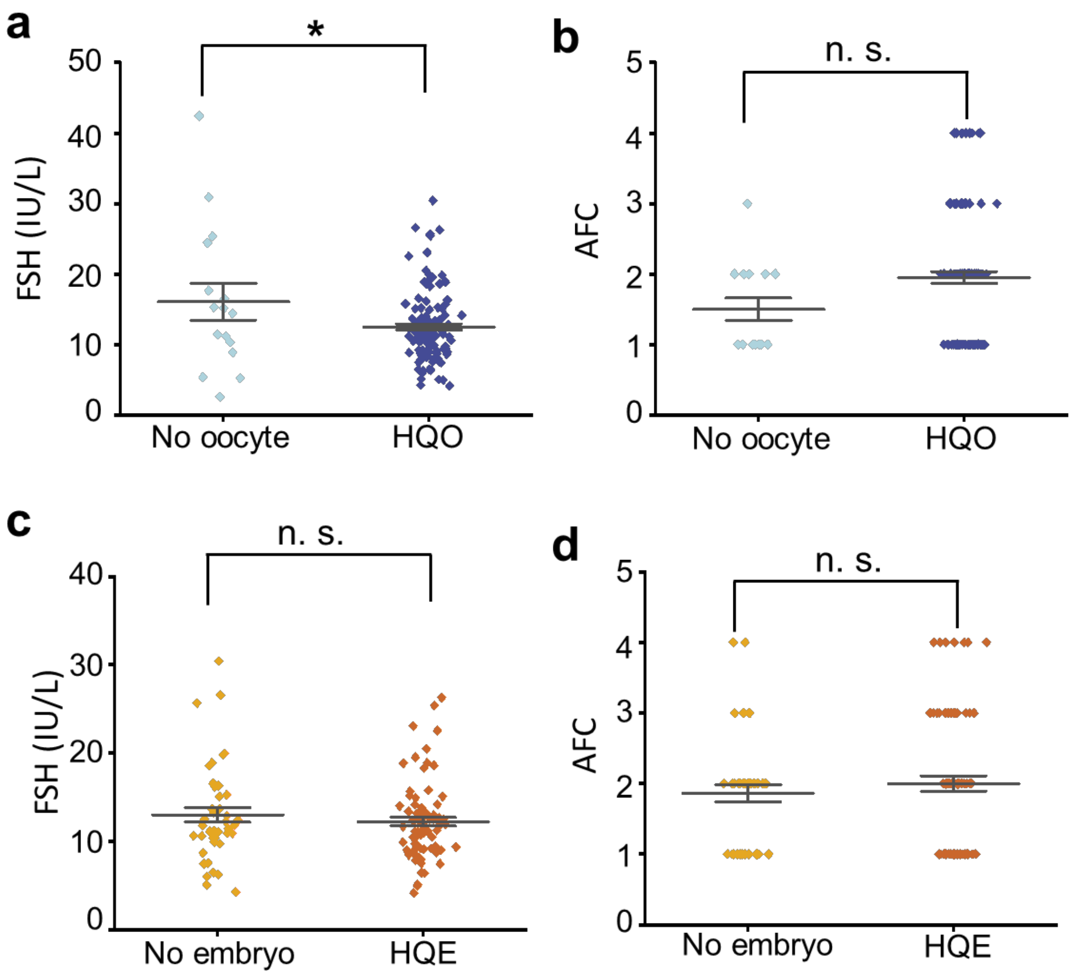

Figure S6 Fertility prediction based on FSH levels, AFC levels. The comparison of probability scores for high-quality oocytes (HQO) group and no oocyte group in predicting oocyte quality based on a) follicle-stimulating hormone (FSH) and b) antral follicular count (AFC) levels. * represented $p < 0.05$ and n. s. represented no significance. The p value was calculated by a two-tailed t-test. The comparison for probability scores of high-quality embryos (HQE) group and no embryo group in predicting embryo quality based on c) FSH and d) AFC levels. The n. s. represented no significance. The p value was calculated by a two-tailed t-test.

Table S1 Parameters of regression equations and limit of detection (LOD) for typical metabolites, including alanine (Ala), arginine (Arg), glucose (Glc), and sucrose (Suc).

Metabolite	Regression equation	R^2	LOD (mg/mL)[1]
Glc	y = 2.2E+05x – 84.3	0.9842	4.14E-05
Suc	y = 2.4E+05x – 207.1	0.9996	1.90E-05
Ala	y = 1.7E+05x +150.8	0.9946	2.77E-05
Arg	y = 1.5E+05x +286.7	0.9873	2.99E-05

1) The signal to noise ratio (S/N) for calculating LOD was 3.

Table S2 The LOD of particles, CHCA, and DHB using 1 μL metabolite solution.

Metabolites	LOD (pmol)		
	NPs	CHCA	DHB
Glc	0.23	55.6	55.6
Suc	0.06	29.2	29.2
Ala	0.31	112.3	112.3
Arg	0.17	5.7	57.4

Table S3 The molecular trapping ratios of particles for typical molecules.

Molecule	Area	Signal intensities of carbon element		Trapping ratio	Mean trapping ratio	p value
		Surface	Background			
Glc	1	28.651	7.116	4.03	5.4	$p < 0.001$
	2	24.618	5.630	4.37		
	3	25.461	3.995	6.37		
	4	27.461	5.016	5.47		
	5	31.297	4.754	6.58		
BSA	1	10.783	4.259	2.53	2.5	
	2	13.714	5.577	2.46		
	3	13.100	4.152	3.16		
	4	7.362	4.561	1.61		
	5	13.210	4.902	2.69		

Table S4 The clinical information of dOR samples.

No.	Age	FSH[1] (IU/L)	AMH[2] (ng/mL)	AFC[3]	HQO[4]	HQE[5]
dOR 1	28	9.73	0.81	2	2	0
dOR 2	26	16.56	N/A	2	4	0
dOR 3	23	18.60	N/A	1	5	4
dOR 4	24	10.38	0.62	3	2	0
dOR 5	25	12.51	N/A	3	12	4
dOR 6	26	26.28	N/A	1	2	1
dOR 7	26	11.23	0.65	1	2	1
dOR 8	27	7.92	0.92	2	2	3
dOR 9	28	9.90	1.09	3	3	1
dOR 10	28	6.25	0.90	1	1	0
dOR 11	28	18.84	0.40	4	1	1
dOR 12	28	15.08	N/A	1	2	1
dOR 13	28	11.11	N/A	3	1	1
dOR 14	29	11.11	N/A	1	1	0
dOR 15	29	5.37	0.27	1	0	0
dOR 16	30	11.83	N/A	2	4	0
dOR 17	30	14.00	1.00	3	6	2
dOR 18	30	11.77	N/A	4	4	0
dOR 19	30	13.41	N/A	3	6	1
dOR 20	31	9.74	0.53	4	1	1
dOR 21	31	25.36	0.35	3	0	0
dOR 22	31	9.18	0.52	2	5	1
dOR 23	31	10.70	1.11	2	6	3
dOR 24	31	8.61	0.27	1	2	2
dOR 25	31	11.88	1.01	3	2	1
dOR 26	31	10.46	0.05	1	1	1
dOR 27	31	8.86	1.06	2	1	1
dOR 28	31	10.62	N/A	2	1	0
dOR 29	31	4.95	0.31	2	3	1
dOR 30	32	12.52	N/A	4	7	4
dOR 31	32	14.42	1.43	1	0	0
dOR 32	32	23.06	1.10	1	2	2
dOR 33	32	7.44	0.99	2	3	3
dOR 34	33	8.68	0.70	1	1	0
dOR 35	33	13.22	0.71	2	2	0
dOR 36	34	12.52	1.14	1	4	2
dOR 37	34	9.36	0.40	4	3	2
dOR 38	34	9.42	1.04	3	3	3
dOR 39	34	12.79	N/A	1	1	1
dOR 40	35	12.70	N/A	3	1	1
dOR 41	35	9.10	0.36	4	2	2

dOR 42	35	18.29	0.25	1	2	1
dOR 43	35	22.54	0.23	1	1	1
dOR 44	35	13.51	1.52	1	4	3
dOR 45	36	5.05	0.17	1	1	0
dOR 46	36	10.98	N/A	2	1	1
dOR 47	36	25.66	N/A	1	1	0
dOR 48	36	7.87	0.53	1	3	3
dOR 49	36	8.45	0.45	3	11	2
dOR 50	36	15.68	N/A	1	1	1
dOR 51	36	24.42	N/A	1	0	0
dOR 52	36	13.18	N/A	2	5	1
dOR 53	36	13.80	N/A	1	2	1
dOR 54	37	30.90	0.25	2	0	0
dOR 55	37	26.55	N/A	2	3	0
dOR 56	37	9.91	0.40	1	2	0
dOR 57	37	12.97	N/A	2	1	1
dOR 58	37	5.22	0.58	2	0	0
dOR 59	38	18.88	N/A	1	1	0
dOR 60	38	15.18	0.48	4	3	1
dOR 61	38	14.15	N/A	3	5	3
dOR 62	38	10.60	N/A	1	2	0
dOR 63	39	8.96	0.14	1	3	3
dOR 64	39	12.36	N/A	1	3	3
dOR 65	39	25.39	N/A	1	2	2
dOR 66	39	15.07	0.78	3	3	0
dOR 67	39	10.96	0.74	2	2	0
dOR 68	39	2.59	0.12	1	0	0
dOR 69	39	7.52	0.80	2	4	4
dOR 70	39	19.96	N/A	2	1	0
dOR 71	40	6.37	0.84	3	7	4
dOR 72	40	12.93	N/A	1	1	1
dOR 73	40	14.17	N/A	4	2	1
dOR 74	40	12.23	0.91	1	2	0
dOR 75	40	16.51	0.49	1	0	0
dOR 76	38	4.24	0.40	2	2	0
dOR 77	40	15.78	N/A	2	2	1
dOR 78	40	10.49	N/A	1	4	2
dOR 79	41	4.14	0.55	1	2	2
dOR 80	41	8.75	0.79	2	5	2
dOR 81	41	6.44	0.68	2	4	1
dOR 82	41	9.21	0.39	3	4	2
dOR 83	36	7.83	0.01	3	4	1
dOR 84	36	19.51	0.69	2	4	1
dOR 85	41	15.27	N/A	1	1	0

dOR 86	41	12.83	N/A	2	2	2
dOR 87	42	12.93	N/A	2	5	1
dOR 88	42	8.19	0.76	2	2	1
dOR 89	42	11.85	N/A	2	1	1
dOR 90	43	13.67	0.95	3	3	0
dOR 91	43	12.90	N/A	2	5	0
dOR 92	43	10.89	N/A	2	3	0
dOR 93	44	11.91	N/A	1	1	1
dOR 94	44	7.53	0.39	2	1	0
dOR 95	45	7.46	0.93	2	3	0
dOR 96	47	13.65	0.56	1	2	0
dOR 97	29	11.49	0.58	1	2	1
dOR 98	30	17.66	0.10	2	0	0
dOR 99	31	11.16	N/A	1	0	0
dOR 100	31	11.10	0.65	2	1	1
dOR 101	32	5.10	0.46	3	2	2
dOR 102	37	9.00	0.97	3	1	1
dOR 103	40	20.49	0.67	3	3	1
dOR 104	35	12.39	N/A	1	2	1
dOR 105	39	11.42	0.44	1	0	0
dOR 106	32	11.13	N/A	2	6	3
dOR 107	34	8.99	0.62	2	1	1
dOR 108	34	12.89	0.16	1	2	1
dOR 109	35	10.56	N/A	2	2	2
dOR 110	40	16.31	N/A	2	1	0
dOR 111	41	14.93	N/A	1	4	2
dOR 112	39	11.42	0.44	1	3	0
dOR 113	41	12.37	N/A	1	3	1
dOR 114	42	12.41	N/A	1	1	0
dOR 115	43	12.35	N/A	1	1	1
dOR 116	44	11.81	N/A	2	4	4
dOR 117	45	6.00	0.43	1	1	0
dOR 118	47	10.39	N/A	4	1	0
dOR 119	49	13.38	0.47	1	1	1
dOR 120	53	16.50	0.50	1	N/A	N/A
dOR 121	49	13.37	0.38	2	3	0
dOR 122	51	19.83	0.48	2	1	0
dOR 123	49	11.22	N/A	2	2	0
dOR 124	47	11.08	N/A	3	3	0
dOR 125	48	18.59	0.15	2	2	0
dOR 126	43	12.50	0.78	3	1	0
dOR 127	43	42.43	N/A	2	0	0
dOR 128	43	10.36	N/A	2	0	0
dOR 129	42	11.68	N/A	3	6	3

dOR 130	41	30.43	N/A	1	1	0
dOR 131	41	12.37	N/A	2	4	3
dOR 132	54	6.47	0.48	2	4	0
dOR 133	54	14.78	N/A	2	N/A	N/A
dOR 134	40	8.21	0.36	2	3	1
dOR 135	51	15.15	N/A	1	0	0
dOR 136	51	15.30	N/A	2	0	0
dOR 137	48	13.15	1.36	1	3	1
dOR 138	49	18.88	1.04	1	1	1
dOR 139	46	8.85	0.32	1	0	0
dOR 140	47	16.16	0.28	2	2	0
dOR 141	45	12.13	0.70	2	1	0

1) FSH represented basal follicle-stimulating hormone.

2) AMH represented antimüllerian hormone. N/A represented that the information was unknown.

3) AFC represented antral follicular count.

4) HQO represented high-quality oocytes.

5) HQE represented high-quality embryos.

Table S5 The clinical information of nOR samples.

No.	Age	FSH[1] (IU/L)	AMH[2] (ng/mL)	AFC[3]
nOR 1	39	5.87	N/A	12
nOR 2	25	6.72	N/A	11
nOR 3	37	9.57	N/A	5
nOR 4	38	6.35	N/A	5
nOR 5	26	8.10	N/A	5
nOR 6	27	6.56	N/A	9
nOR 7	43	8.31	N/A	5
nOR 8	27	6.81	4.94	2
nOR 9	27	6.73	N/A	5
nOR 10	28	8.67	N/A	9
nOR 11	28	6.25	N/A	12
nOR 12	28	6.69	N/A	6
nOR 13	28	7.93	N/A	5
nOR 14	34	6.97	N/A	5
nOR 15	29	5.25	N/A	7
nOR 16	29	8.63	3.33	11
nOR 17	29	9.34	N/A	12
nOR 18	29	9.59	8.96	12
nOR 19	30	7.89	N/A	8
nOR 20	30	5.90	N/A	9
nOR 21	30	9.16	N/A	6
nOR 22	30	7.52	2.03	7
nOR 23	30	5.87	N/A	7
nOR 24	30	8.38	N/A	11
nOR 25	30	8.65	N/A	6
nOR 26	30	8.05	N/A	12
nOR 27	30	6.22	N/A	7
nOR 28	35	7.09	6.21	9
nOR 29	30	9.58	N/A	10
nOR 30	31	4.05	N/A	12
nOR 31	31	6.59	N/A	7
nOR 32	31	3.84	N/A	12
nOR 33	31	5.18	N/A	12
nOR 34	31	6.73	N/A	5
nOR 35	31	7.39	N/A	11
nOR 36	31	8.32	N/A	11
nOR 37	31	7.18	N/A	12
nOR 38	35	5.86	N/A	6
nOR 39	31	5.51	N/A	11
nOR 40	31	9.17	N/A	10
nOR 41	31	6.38	N/A	5

nOR 42	31	6.07	N/A	8
nOR 43	33	6.06	N/A	6
nOR 44	32	7.34	2.55	7
nOR 45	32	5.81	N/A	7
nOR 46	32	5.71	N/A	10
nOR 47	33	5.73	N/A	9
nOR 48	32	7.14	N/A	8
nOR 49	32	7.27	N/A	7
nOR 50	32	7.64	N/A	9
nOR 51	32	7.19	N/A	11
nOR 52	32	6.50	N/A	12
nOR 53	32	6.20	N/A	6
nOR 54	32	5.70	N/A	7
nOR 55	32	6.71	N/A	12
nOR 56	32	8.55	N/A	8
nOR 57	32	5.15	N/A	11
nOR 58	32	7.32	N/A	12
nOR 59	33	3.81	N/A	7
nOR 60	33	5.63	N/A	6
nOR 61	33	7.90	N/A	5
nOR 62	33	6.49	3.98	9
nOR 63	33	6.78	N/A	12
nOR 64	33	7.47	N/A	7
nOR 65	33	9.26	N/A	6
nOR 66	33	6.58	N/A	12
nOR 67	33	6.82	N/A	5
nOR 68	33	6.68	N/A	9
nOR 69	33	7.98	N/A	11
nOR 70	33	7.07	N/A	5
nOR 71	33	6.06	N/A	6
nOR 72	34	6.82	N/A	6
nOR 73	34	8.36	N/A	9
nOR 74	34	3.78	N/A	5
nOR 75	34	7.95	N/A	12
nOR 76	34	6.06	N/A	10
nOR 77	34	8.08	N/A	12
nOR 78	34	6.72	N/A	12
nOR 79	34	6.38	1.92	5
nOR 80	34	6.04	N/A	5
nOR 81	34	5.84	N/A	9
nOR 82	34	6.62	N/A	5
nOR 83	34	8.19	N/A	12
nOR 84	34	5.93	N/A	8
nOR 85	34	6.04	N/A	6

nOR 86	34	6.47	N/A	7
nOR 87	34	9.57	N/A	8
nOR 88	34	5.14	N/A	12
nOR 89	34	9.26	N/A	5
nOR 90	35	6.76	N/A	11
nOR 91	35	6.67	N/A	8
nOR 92	35	8.72	N/A	8
nOR 93	35	7.96	N/A	12
nOR 94	35	6.91	N/A	7
nOR 95	35	6.52	N/A	6
nOR 96	35	4.27	N/A	8
nOR 97	35	4.84	N/A	6
nOR 98	35	6.48	N/A	6
nOR 99	36	7.84	N/A	5
nOR 100	36	6.93	N/A	9
nOR 101	36	4.93	N/A	6
nOR 102	36	5.57	N/A	10
nOR 103	36	7.82	N/A	9
nOR 104	36	5.30	N/A	8
nOR 105	36	5.58	N/A	7
nOR 106	36	5.52	N/A	12
nOR 107	37	2.93	N/A	12
nOR 108	37	9.13	3.29	4
nOR 109	37	7.34	N/A	11
nOR 110	37	7.56	1.29	4
nOR 111	37	6.89	N/A	10
nOR 112	37	8.47	N/A	5
nOR 113	37	6.36	N/A	12
nOR 114	37	7.38	N/A	9
nOR 115	37	5.99	N/A	6
nOR 116	37	2.73	N/A	12
nOR 117	38	7.45	N/A	7
nOR 118	38	5.70	N/A	9
nOR 119	38	7.00	N/A	8
nOR 120	38	2.22	N/A	11
nOR 121	39	9.47	N/A	12
nOR 122	39	7.25	N/A	5
nOR 123	39	9.94	N/A	7
nOR 124	39	3.33	N/A	12
nOR 125	40	6.33	N/A	8
nOR 126	40	3.41	N/A	12
nOR 127	40	9.70	N/A	7
nOR 128	40	8.82	N/A	5
nOR 129	40	5.96	N/A	6

nOR 130	40	6.55	N/A	6
nOR 131	42	9.87	5.52	9
nOR 132	42	6.85	N/A	5
nOR 133	43	8.35	N/A	5
nOR 134	43	9.25	N/A	5
nOR 135	43	7.43	N/A	10
nOR 136	44	6.24	3.74	6
nOR 137	40	4.94	N/A	7
nOR 138	40	6.02	N/A	12
nOR 139	25	8.92	N/A	6
nOR 140	27	6.17	N/A	8
nOR 141	36	7.50	N/A	7
nOR 142	36	9.88	N/A	6
nOR 143	36	5.16	N/A	6
nOR 144	37	7.31	N/A	6
nOR 145	34	4.60	N/A	8
nOR 146	34	8.59	N/A	5
nOR 147	34	6.75	N/A	7
nOR 148	34	6.74	N/A	7
nOR 149	33	6.42	1.15	4
nOR 150	32	3.70	N/A	7
nOR 151	22	7.82	N/A	6
nOR 152	26	4.06	N/A	10
nOR 153	28	5.80	N/A	12
nOR 154	29	6.75	4.4	5
nOR 155	29	7.84	N/A	5
nOR 156	30	5.67	N/A	5
nOR 157	30	7.50	N/A	5
nOR 158	30	6.09	N/A	9
nOR 159	30	6.20	N/A	5
nOR 160	31	8.30	N/A	7
nOR 161	31	7.11	N/A	12
nOR 162	31	9.20	N/A	11
nOR 163	31	9.37	N/A	7
nOR 164	32	6.45	N/A	12
nOR 165	32	7.52	N/A	9
nOR 166	31	5.30	N/A	12
nOR 167	29	5.52	N/A	7
nOR 168	30	3.21	N/A	7
nOR 169	22	8.72	N/A	8
nOR 170	26	6.88	N/A	12
nOR 171	26	5.96	N/A	6
nOR 172	27	5.78	N/A	11
nOR 173	27	6.70	N/A	12

nOR 174	25	5.62	N/A	7
nOR 175	27	7.51	N/A	6
nOR 176	28	7.42	N/A	6
nOR 177	28	6.17	N/A	6
nOR 178	29	5.47	N/A	9
nOR 179	29	6.08	N/A	7
nOR 180	29	7.87	N/A	9
nOR 181	26	6.07	N/A	5
nOR 182	30	9.74	N/A	9
nOR 183	31	7.81	N/A	8
nOR 184	31	9.29	4.71	7
nOR 185	32	9.89	N/A	10
nOR 186	32	5.62	N/A	8
nOR 187	26	7.07	N/A	12
nOR 188	27	6.93	N/A	6

1) FSH represented basal follicle-stimulating hormone.

2) AMH represented antimüllerian hormone. N/A represented that the information was unknown.

3) AFC represented antral follicular count.

Table S6 The age match of dOR and nOR samples in the discovery cohort.

	Category	dOR	nOR	p value[1]
Discovery cohort	Age (SD, year)	35.15 (5.11)	34.10 (4.07)	0.06

1) The p value was calculated by a two-tailed t-test.

Table S7 The model performance for dOR diagnosis using ridge regression (RR), neural network (NN), support vector machine (SVM), and random forest (RF).

Algorithms	Accuracy (Acc)	Sensitivity (Sen)	Precision (Pre)	F1 score[1] (F1)
RR	0.829	0.775	0.796	0.785
NN	0.800	0.649	0.818	0.724
SVM	0.767	0.541	0.822	0.652
RF	0.764	0.613	0.756	0.677

1) The F1 scores were calculated by the harmonic mean of sensitivity and precision.

Table S8 Model performance of the area under the curve (AUC) for dOR diagnosis in the discovery and independent validation cohorts.

Algorithms	Discovery cohort (95% CI)[1]	Validation cohort (95% CI)
RR	0.905 (0.870-0.940)	0.929 (0.867-0.991)
NN	0.856 (0.812-0.900)	0.924 (0.855-0.993)
SVM	0.847 (0.801-0.893)	0.905 (0.833-0.978)
RF	0.796 (0.744-0.849)	0.874 (0.784-0.963)

1) CI represent confidence interval.

Table S9 Biomarker validation using FT-ICR-MS.

Metabolites	Molecular formula	Theoretical m/z value	Mass error (ppm)[1]	p value[2]
Lactic acid (LA)	$C_3H_6O_3$	135.0029	5.19	2.04E-24
Succinic acid (SA)	$C_4H_6O_4$	156.9898	1.97	5.86E-08
Pyruvic acid (PA)	$C_3H_4O_3$	164.9351	1.64	4.55E-02
Glucose (Glc)	$C_6H_{12}O_6$	203.0526	3.20	8.29E-04
Fructose 6-phosphate /Glucose 6-phosphate (F6P/G6P)	$C_6H_{13}O_9P$	283.0189	0.94	3.54E-16

1) The ppm value was calculated according to the measured and theoretical values of m/z feature.

2) The p value was calculated using the average intensity between the dOR and nOR groups by two-tailed t-test.

Table S10 Biomarker validation by LC MS.

Metabolites	Molecular formula	Detection mode	Theoretical m/z value	Retention time (min)	Mass error (ppm)[1]
LA	$C_3H_6O_3$	Negative mode	89.02	2.99	-0.02
SA	$C_4H_6O_4$	Negative mode	117.02	6.97	1.26
PA	$C_3H_4O_3$	Negative mode	87.01	2.18	-0.31
Glc	$C_6H_{12}O_6$	Negative mode	179.06	3.60	1.51
F6P/G6P	$C_6H_{13}O_9P$	Negative mode	259.02	8.12	-2.16

1) The ppm value was calculated according to the measured and theoretical values of m/z feature.

Table S11 Biomarker panel constructed for dOR diagnosis.

Metabolites	p value[1]	AUC (95% CI)
LA	3.00E-4	0.765 (0.716-0.815)
SA	< 4.00E-13	0.571 (0.510-0.632)
PA	4.63E-13	0.599 (0.539-0.660)
Glc	2.34E-9	0.637 (0.577-0.696)
F6P/G6P	2.70E-4	0.803 (0.756-0.850)
Panel	/	0.849 (0.809-0.890)

1) The p value was calculated by the AUCs between single biomarker and biomarker panel through Delong test.
